# Retaining Those Who Care: Ethical Climate, Leadership, and Workforce Sustainability in Healthcare

**DOI:** 10.3390/healthcare13233014

**Published:** 2025-11-21

**Authors:** Ana Poeira, Lucília Nunes

**Affiliations:** 1 Escola Superior de Saúde, Instituto Politécnico de Setúbal, 2910-761 Setúbal, Portugal; lucilia.nunes@ess.ips.pt; 2 Comprehensive Health Research Centre (CHRC)—Polo NMS, 1169-056 Lisboa, Portugal

**Keywords:** ethical leadership, healthcare systems, moral courage, leader conscientiousness, personnel turnover, retention, workforce sustainability

## Abstract

**Background/Objectives:** The global shortage of healthcare professionals has exacerbated ethical and organizational challenges that threaten the sustainability of health systems. High turnover rates, combined with the emigration and attrition of qualified nurses, make it essential to understand how institutional factors affect professional well-being and workforce retention. This essay examines how organizational ethics—particularly ethical climate, organizational justice, and ethical leadership—influence healthcare professionals’ experiences and their intentions to remain in or leave the sector. **Methods:** The discussion adopts a conceptual and reflective approach, drawing on key theories and empirical findings from the literature on organizational ethics and turnover. It explores the relationships between ethical environments, professional recognition, and moral well-being within healthcare organizations. **Results:** Evidence suggests that ethical and fair organizational climates strengthen trust, professional commitment, and long-term retention. Conversely, perceptions of injustice, lack of recognition, or moral distress tend to increase dissatisfaction and the intention to leave the profession. **Conclusions:** The essay underscores ethics as a central component of workforce sustainability in healthcare and calls for leadership and policy actions that move beyond individual coping strategies toward systemic ethical practices promoting fairness, recognition, and well-being at work.

## 1. Introduction

The sustainability of healthcare systems largely depends on their ability to attract and retain qualified, motivated, and ethically committed healthcare professionals, with professional retention constituting a true pillar of sustainability. This essay focuses on the ethical foundations of workforce retention, arguing that moral and institutional factors are as decisive as economic or managerial ones.

In recent years, external turnover among healthcare professionals has risen sharply, driven by factors such as work overload, burnout, emotional exhaustion, perceptions of organizational injustice, team-level psychosocial dynamics, and moral distress [1,2,3,4]. The impact is far-reaching: it threatens continuity of care, reduces quality and patient safety, and weakens interprofessional cohesion [5,6]. A recent systematic confirmed that unfinished nursing care is strongly associated with burnout, dissatisfaction, intention to leave, and turnover, underscoring its role as a critical driver of workforce instability [7].

To illustrate this point, the film Late Shift [8], directed by Petra Volpe, portrays the night shift of Floria, a nurse in a chronically understaffed hospital, who faces a set of ethical, emotional, and professional demands without institutional support. The narrative, praised by critics for highlighting the tensions between vocation, suffering, and organizational responsibility, presents a fictional yet realistic reflection of the systemic implications of the erosion of managerial ethics and the devaluation of healthcare professionals [9].

Despite the greater visibility now given to professional well-being, organizational discourse often continues to shift responsibility onto individuals, encouraging personal resilience strategies while neglecting structural and institutional reforms that would favor retention. Within this context, organizational ethics emerges as a crucial lens for examining how institutions can promote—or erode—fair, safe, and supportive work environments [1,10,11,12]. The novelty of this essay lies in its integrative ethical perspective, positioning organizational ethics not merely as a normative framework, but as a strategic determinant of professional retention. Although empirical studies on turnover frequently include variables such as ethical climate, justice, and leadership among organizational predictors, their ethical significance is often overlooked. This reflection argues that these dimensions may exert a deeper, more enduring influence on professionals’ retention than traditionally emphasized factors, such as remuneration.

Drawing on organizational ethics frameworks such as ethical climate theory [10], organizational justice [11], and ethical leadership [12], this article proposes a critical conceptual reflection on the relationship between institutional practices, professional well-being, and external turnover. It argues that practice environments are moral spaces in which institutional decisions shape professionals’ well-being, motivation, and intentions to remain or to leave. The novelty of this essay lies in its integrative ethical perspective, positioning organizational ethics not merely as a normative framework, but as a strategic determinant of professional retention.

This concept paper thus aims to contribute to the debate on the ethical imperative of professional retention, challenging the fragmentation between clinical ethics and organizational ethics. It argues that retaining caregivers is not merely a human resource management strategy, but a collective ethical responsibility, a condition for high-quality care, and an imperative for the sustainability of healthcare systems.

The conceptual framework adopted in this essay draws on three complementary constructs. Ethical climate refers to the shared perceptions of what constitutes ethically appropriate behavior within an organization, shaping moral reasoning and decision-making in the workplace [10,13]. Organizational justice concerns the perceived fairness of procedures, interactions, and outcomes, encompassing distributive, procedural, and interactional dimensions that influence trust, satisfaction, and commitment [11]. Ethical leadership embodies the moral dimension of managerial behavior, modeling fairness, transparency, and care, and guiding employees’ ethical conduct through example and moral influence [12,14,15].

Grounded in this conceptual framing, the following research questions guide the reflection developed in this essay: How do organizational ethical climates influence healthcare professionals’ intention to remain in or leave the profession? In what ways do perceptions of organizational justice and ethical leadership shape professional well-being and moral commitment? How can organizational ethics be reframed as a strategic axis for workforce sustainability in healthcare?

## 2. Literature Review

The literature review addresses the conceptual foundations underlying professional retention in healthcare, emphasizing the ethical and organizational dimensions that influence professionals’ motivation to remain in or leave their roles. This section is organized into four thematic parts: the meaning and value of work, the specific challenges of the healthcare sector, the role of organizational ethics and ethical leadership, and the integration of these dimensions in promoting workforce sustainability. Together, these perspectives provide a theoretical framework for understanding retention as both an ethical and systemic responsibility.

### 2.1. Why Do People Work?

Understanding professionals’ intention to remain in or leave their professions requires first exploring the meaning and value attributed to work. These dimensions shape motivation, engagement, and ultimately the ethical basis of professional retention.

All human existence has as its first premise the fact that human beings, to act as subjects of history, must be able to live. To live, one needs food, drink, shelter, clothing, and a few other essentials. The satisfaction of basic needs occurs through the production of use values, which is the first historical act, work.

According to Lukács, work emerges as a means of the struggle for existence, enabling the transition from one level of being—the merely biological—to another, qualitatively different one—the social being—that is, the passage from the condition of animality to the condition of humanity. Therefore, work’s mediating nature between necessity, the subject of that necessity, and nature. “For only in this way can our work embrace both the demands of the day and the creation of the future” (p. 92) [16].

The term “work” assumes specific meanings across contexts, yet its lowest common denominator is the practice through which people contribute to their families, communities, or societies. It is crucial recognize that the overwhelming majority of the world’s population depends on work for their subsistence, that is, they are “a huge class-that-lives-off-work”.

Work is a central part of both our personal and social identities, as it is both an individual pursuit and a way to connect with our communities. Therefore, the concept of work extends beyond paid employment to include other valuable contributions. Tasks such as caring for children or the elderly are an extremely important form of labor that contributes significantly to the well-being and success of our society.

It is also essential, in this context, to distinguish between work and employment. Work is a broader concept, encompassing all human activity that produces value, whether material or immaterial, including unpaid activities such as family care, volunteering, or other forms of social contribution. Employment, on the other hand, refers to a specific form of work, characterized by a contractual relationship, remuneration, and legally regulated rights. Reducing the concept of work to mere employment makes invisible the fundamental contributions that sustain society and ensure the collective quality of life. This distinction is crucial when considering the reasons why people work and how they find satisfaction, as both paid and unpaid work influence identities, social relationships, and the perception of meaning.

When we use the term “employment” as a synonym for “work”, we risk rendering invisible the multiple dimensions of human activity that are essential to individual and collective well-being. Moreover, such a reduction carries the danger of aligning with a Taylorist view of labour, in which human capacities are subordinated to efficiency, control, and output, neglecting the social, affective, and ethical aspects that make work meaningful.

Work are shaped not only by the nature of the work itself or its value in our society, but also by the political and economic structures of our societies. These structures play a fundamental role in shaping the future of work, not only by influencing how much people earn and how their rights and interests are protected, but also by influencing the likelihood of people finding meaning in their work.

At its core, we work because we need to earn a living.

Furthermore, we can feel engaged in our work, satisfied, and challenged. Satisfied people do their work because they feel in control, have autonomy, and learn new things, developing as professionals and as persons. Work also offers an opportunity for social engagement, a space for social interactions, as many tasks are performed collaboratively within teams. Ultimately, these individuals are satisfied with their work because they consider their tasks meaningful. Potentially, they know that their work makes a difference. For example, a nurse or a teacher can provide care in meaningful ways and improve the lives of others. These diverse sources of job satisfaction raise some questions. And it is on these aspects of (dis)satisfaction at work and retention in context that we will reflect.

In December 2018, the European Group on Ethics in Science and New Technologies (EGE) was asked by the European Commission to explore the ethical and societal questions linked to the future of work. Both professional life and society are being reshaped by globalization, demographic evolution, and the swift advancement of technology. The resulting report, “Future of work, future of society” [17], highlights the need to rethink the current social contract to uphold European values such as human dignity, solidarity, and justice. As stated in the document, “When work is both decent and meaningful, we are typically embedded in joint action with fellow human beings, ideally pursuing common goals, for shared reasons and enjoying the satisfaction of achievement, recognition, and self-improvement. We exercise basic human capacities in the process, as well as physical, psychological (both emotional and cognitive), and social ones” (p. 56) [17].

Beyond the fact that most of us must earn a living, a variety of motivations and goals determine why and how we work. Beyond the necessity of earning a living, work is shaped by multiple motivations and goals—economic, social, and ethical. Understanding these dimensions is essential for addressing professional retention as a strategic priority for sustainable healthcare systems.

People who find satisfaction in their jobs are often motivated by a range of factors. These can include a sense of personal fulfillment, the opportunity to exercise responsibility, receiving appreciation from others, and the chance to develop their skills and creativity. They may also be driven by a desire to contribute to their company’s success and their own well-being, to benefit society and others, to learn new things, and to engage in social interaction—all of which ultimately lead to a feeling of doing something meaningful. These elements underscore work’s inherent worth, the value we get from doing the tasks themselves, regardless of external incentives like getting paid or advancing a career.

These intrinsic aspects of work are crucial for viewing one’s life as a logical and developing story, which is fundamental to self-respect and the formation of a personal identity. Although work offers both intrinsic and instrumental value for most people, the importance of each of these values varies from person to person.

In conclusion, people work for a combination of practical, psychological, affective, and social reasons. It is not just about earning a paycheck, though that is a big part of it. Work can be a matter of survival and security, stability, sense of meaning (purpose), and fulfilment, personal growth. Additionally, one can tailor their identity and self-worth, develop a community and social environment, gain recognition, and contribute to society.

Contemporary professionals increasingly recognize that lifelong employment in the same role is neither mandatory nor desirable. Opportunities for mobility, career change, and sectoral transitions are now widely accepted, and, ultimately, many prefer leaving a harmful work environment rather than enduring it.

Hospitals and organizations that fail to acknowledge this reality lag behind in implementing strategies that promote retention and well-being.

In the healthcare context, the meaning attributed to work has a profound impact on professionals’ commitment and intention to remain in the profession. When the moral and relational meaning of care is eroded, through organizational injustice, lack of recognition, or moral distress, professionals may experience a loss of purpose and disengagement that lead to external turnover. Conversely, when nurses and other healthcare professionals perceive their work as meaningful and aligned with their ethical values, they are more likely to demonstrate resilience, commitment, and a sustained desire to continue in the profession. This connection between meaningful work, ethical climate, and retention has been emphasized in recent studies highlighting that professional fulfillment and moral well-being are among the strongest predictors of intention to stay in healthcare settings [18,19,20].

In summary, understanding why people work provides the ethical and motivational foundation for reflecting on satisfaction, recognition, and the decision to stay or leave, central aspects of workforce sustainability in healthcare.

### 2.2. The World of Work and the Value of Recognition—What Am I Doing Here?

In the world of work, uncertainties, resistance, difficulties, obstacles, incidents, and accidents are always present. Therefore, it is pertinent to note that work often involves adapting, adjusting, or creating alternatives to overcome the challenges posed by new situations.

According to Dejours, within organizations, work depends on two key factors: coordination (which implies the use of power over others, as a form of domination) and cooperation (which implicitly implies a specific form of conviviality) [21].

Dejours expresses a curious idea that there is a kind of curse on what lies at the heart of work. Affective reactions to resistance to reality and failure are not visible. Irritation, discouragement, and doubts about one’s own competence are hidden. Suffering, in general, does not belong to the visible world. Suffering, like all affects and feelings, like all subjectivity, is experienced with one’s eyes closed.

People generally do not work alone. They work for a boss, with colleagues, or with subordinates. Working together is extremely complicated, as each person charts their own path, invents their own discoveries, and builds their own knowledge. And disorder, if not chaos, inevitably arises. Here, on the collective level, we find a gap, a separation between the injunctions to work together—what is called coordination—and what those who can actually work together do—cooperation. The gap between coordination and cooperation can be enormous.

In exchange for the contribution people make to the organization of work, the company, or society, they expect remuneration. This remuneration is, obviously, and first and foremost, material: salary, fees. However, it is easy to see that what mobilizes intelligence and zeal, both individual and collective, is not only the material dimension of remuneration but also the symbolic dimension. What people expect in exchange for their involvement and suffering is a moral reward that takes an exact form: recognition [22].

The judgment of recognition refers specifically to the work performed: its usefulness on the one hand, its quality on the other. Only when I receive recognition for the usefulness and quality of my work do I experience the intense satisfaction of my relationship with work. When others recognize the quality of my work, then I can shift that recognition from the profile of doing to the profile of being: I am more intelligent, more competent, more self-assured after work than before. I develop, my identity is strengthened, I flourish, and I find fulfillment.

This recognition can also be interpreted through the lens of the psychological contract, which is the set of unwritten expectations that reciprocally bind the employee and the employer [23]. The idea of reciprocity in the relationship between workers and organizations has long been discussed in organizational studies. At its core, this perspective highlights an exchange dynamic: employees contribute effort, loyalty, and commitment, in return for which they expect recognition, support, and appreciation from the organization [24]. Even when the term “psychological contract” is not explicitly mentioned, the concept rests on this very logic of implicit obligations and shared expectations [24]. When individual and collective contributions are acknowledged, the psychological contract is strengthened, which in turn nurtures engagement, motivation, and a sense of belonging that extends well beyond material rewards. Conversely, the absence of recognition represents a breach of this implicit contract, negatively affecting satisfaction, mental health, and professional retention [1,3,25,26,27,28].

In the healthcare sector, breaches of the psychological contract, such as lack of organizational support, perceived injustice, or misalignment between professional values and managerial priorities, can have significant repercussions on professional well-being [26]. Such violations often manifest as moral distress, emotional exhaustion, and a sense of disillusionment, which may ultimately lead to withdrawal or turnover [29]. Conversely, when the psychological contract is fulfilled through recognition, fairness, and ethical leadership, it fosters professional satisfaction, engagement, and a sustained sense of moral integrity at work. These dynamics are particularly relevant in nursing, where well-being is closely tied to ethical congruence and perceived organizational justice [27,30,31].

The world of work is far from ideal, and specific work organizations tend to systematically undermine the dynamic between contribution and reward. They disrupt the conditions of recognition and cooperation, undermining the foundations of working together. We want to emphasize that how work is organized can and should be questioned—even if it is traditional, it is not the result of fate or destiny. Every work organization is a human construct and only develops with the consent and collaboration of people.

Currently, a significant proportion of workers report disengagement or even suffer from, their work. Many experience disconnection from their own work, giving relevance to issues surrounding the balance between work and private life (perhaps explaining the tenacity of the term “work–life balance” which suggests that work and life are mutually exclusive). We now know that work has an extraordinary influence on individuals’ mental health. Workers are never passive subjects in the face of the constraints imposed by work organization. This is why work can hardly be considered a neutral element in mental health [32].

Disconnected, actively disengaged, and unhappy with their work. What are the consequences overall, and in the healthcare sector in particular?

### 2.3. Specificities in the Health Sector and the Phenomenon of Turnover: “Should I Stay, or Should I Go?”

“Should I Stay or Should I Go” is a song by British rock band The Clash, from their album Combat Rock, written in 1981 and features Mick Jones on lead vocals. It became the band’s only number one single in the UK, a decade after its original release.

The healthcare sector currently presents an enormous challenge for several reasons that converge and reinforce one another. There is a global shortage of health professionals, which seriously compromises universal health coverage and has been consistently reported across 204 countries and territories [33]. This shortage is also confirmed by OECD data, showing persistent deficits and widening gaps in health workforce availability [34]. This shortage of health professionals, estimated to reach almost 18 million by 2030 [35], has been worsening since 2021, according to the Federation of European Social Employers [36].

In addition to the global deficit, there is a marked inequality in the geographical distribution of professionals: low- and middle-income countries are disproportionately affected, while in high-income contexts, the shortage is intensified by migration flows, the aging of the workforce, and early exits from the profession [35].

Additionally, more than 20% of professionals frequently consider leaving their jobs due to stress, difficulties in balancing their personal and professional lives, and low job satisfaction [37].

In addition to a shortage of professionals, it is difficult to attract new healthcare professionals, such as nurses, because younger generations often find the profession unattractive due to low salaries and professional status [38]. In fact, the interest of young people in healthcare professions declined in about half of OECD countries between 2018 and 2022, according to the OECD 2025 report “What do we know about young people’s interest in health careers?” This trend creates a dual challenge: newly graduated professionals often abandon the profession early, while more experienced workers accelerate retirement or seek alternative careers after losing hope for meaningful change [39,40].

The COVID-19 pandemic had a paradoxical impact—initially, it generated increased interest in professions that have saved millions of lives. Still, this interest eventually faded as the highly demanding working conditions and low pay for workers became more apparent. Given the circumstances, we now face not only scarcity and high turnover but also abandonment and loss of generational renewal, a scenario that disperses capabilities and dilutes skills trained in specific clinical contexts.

Given the circumstances, we will have to face the predicted lack of interest, scarcity, and abandonment, in addition to high mobility, which disperses capabilities and dilutes skills trained in specific clinical contexts.

We have known for some time, from studies spanning over a decade, that there is a link between staff turnover and negative patient outcomes, such as increased errors [41] and longer hospital stays [42]. This also leads to lower patient satisfaction [43].

When turnover becomes frequent, the cohesion and continuity of healthcare teams are compromised, generating organizational instability and reduced job satisfaction [44]. Over time, this dynamic may intensify professionals’ intentions to leave the profession, leading to a significant depletion of experiential knowledge and collective competence in the sector [45].

We have highlighted a meaningful relationship between professionals’ exit choices and the organizational impact, including a loss of expertise and increased training effort for new professionals, among other consequences [46]. For example, it is estimated that the cost of each individual turnover is three times the salary of a nurse [47].

The consequences of turnover extend far beyond staff changes, affecting the stability and functioning of healthcare organizations. To understand this process, it is essential to differentiate between turnover intention and actual turnover. The first, often described as the intention to leave, reflects a worker’s deliberate thought or plan to resign within a defined period. Drawing on the Theory of Planned Behavior [48], turnover intention is viewed as the stage that precedes actual withdrawal from employment, generally beginning with dissatisfaction and culminating in the decision to exit [49]. In contrast, actual turnover refers to the employees’ concrete departure from the organization. From a management perspective, one of the most common indicators of this process is the turnover rate, which measures the proportion of workers who resign during a given period, typically calculated annually relative to the total number of employees. This metric offers a quantitative view of workforce stability [50].

Naturally, turnover can be voluntary or involuntary and may be avoidable or inevitable. It can also mean completely leaving the department, or the organization, or the profession [51]. In this case, turnover turns into abandonment of the profession.

Beyond the individual perspective, and while some degree of turnover is expected and beneficial to an organization, it is essential to find a balance and develop strategies to retain qualified and competent professionals. For example, in nursing, a turnover rate of n 5–10% is considered adequate [52].

Efforts to address employee turnover should consider how complex and multifactorial this phenomenon is. Both turnover intention and actual turnover emerge from the interaction of individual, team, and organizational influences. Studies have highlighted a range of individual aspects, such as workload, autonomy, and demographic characteristics, while also noting that team-related and managerial factors, including staffing levels and leadership approaches, strongly shape professionals’ decisions to stay or leave. Over the past decades, researchers have proposed several conceptual frameworks to clarify these interconnections and to explain turnover within healthcare settings [6,53].

Although sociodemographic and contextual factors (such as age, professional experience, or workload) influence turnover intention, this essay privileges a conceptual understanding of how such variables are mediated by ethical and organizational conditions, particularly perceptions of justice, recognition, and moral climate. These ethical dimensions provide the interpretive framework through which individual and contextual characteristics acquire meaning within professional retention.

Understanding these factors could ultimately lead to the identification of ways to improve retention [54,55] and mitigate turnover. Various interventions have been developed and implemented, including strategies to improve working conditions, provide professional development opportunities, and foster a positive organizational culture—and it has become clear that the effectiveness of these can vary across contexts and the specific challenges faced by healthcare professionals in different settings [56,57].

A recent comprehensive review synthesized evidence from 37 systematic reviews, encompassing 511 primary studies that examined staff turnover in the health and social care sectors. The majority of these studies focused on healthcare professionals, particularly nurses [58]. This large-scale synthesis identified a broad range of determinants, categorized into three main domains: sociodemographic characteristics, work environment features, and aspects of personal attitudes and functioning. Although several interventions have been designed to reduce turnover, the overall strength of the evidence supporting their effectiveness remains limited. Methodological weaknesses, potential biases, and contextual variability between studies hinder generalization, suggesting that the success of such interventions may depend heavily on specific organizational and cultural settings [58].

Age and level of professional experience provide mixed evidence as antecedents of turnover intention and/or actual turnover. One explanation may be the “reality shock” upon entering the profession—although new graduates are familiar with the health care environment through clinical experience during their degree, it can be more overwhelming than expected, causing them to leave their jobs and the profession early on—a mechanism like what is known as “praxis shock” [59]. On the other hand, more experienced professionals who have invested considerable time in their careers may be more likely to leave the profession, for example, because they lose hope that things will change [60].

Team-level psychosocial aspects, such as social support, team cohesion, and perceived fairness, can influence turnover intentions. Effective leadership, in particular, can mitigate stress and provide a supportive work environment, thereby reducing turnover intentions [61]. Recent evidence also suggests that promoting ethics education within healthcare institutions contributes to a stronger ethical climate, which in turn enhances professional satisfaction and retention [62].

We know that a sense of belonging and support within a team can mitigate stress in health environments [63]. Conversely, a negative organizational culture (characterized by poor communication, lack of trust, and limited employee engagement) can contribute to higher turnover rates [64].

Finally, we should add another idea to this complex issue—the reality of nurses changing profession and new graduates abandoning (and changing) profession should worry us, and we should recognize that it relates to incomes. In her study on external turnover in Nursing, Poeira found that professionals should acknowledge that the “Opportunity to increase income” was one of the most commonly mentioned factors by nurses as contributing to their decision to change organization, revealing that nurses seek salaries that are compatible with the high complexity and responsibility of their work [65].

The greater the professional satisfaction, autonomy, and recognition, the lower the nurses’ probability of leaving the nursing profession, and consequently, the lower the external turnover rates [39].

These empirical findings call for (or demonstrate) the idea of fairness and ethical leadership.

### 2.4. Organizational Ethics and Ethical Leadership—Why Do I Desire an Ethical Leader?

The choice of “ethical leadership” in this section brings us closer to Brown & Treviño’s theory [12] as a starting point. But it also intersects with Kenneth Blanchard and Norman Peale’s conceptualization of ethical management and the work of Jennifer Prah Ruger related to social justice [66,67].

The literature highlights the significance of various psychological factors as mediators in understanding employee turnover intentions within a specific leadership structure. For example, job satisfaction, commitment, citizenship behavior, and intrinsic motivation have been identified as psychological factors that mediate between a leadership style and turnover intentions [31,33,68,69].

An ethical leadership style in management can positively increase employees’ intrinsic motivation and, consequently, buffer the negative effect of turnover; however, its fundamental role has been little highlighted in the healthcare context, despite robust recent evidence confirming that ethical climate strongly influences job satisfaction, well-being, and professionals’ intention to remain in healthcare organizations [13].

Ethical leadership serves as a protective mechanism for the psychological contract established between the employee and the organization. When leaders demonstrate integrity, transparency, and fairness, they reinforce the perception of implicit reciprocity that sustains this contract, thereby strengthening organizational trust and fostering a sense of psychological safety [70], which is particularly relevant in contexts of high moral distress, such as during the COVID-19 pandemic, where perceived organizational support proved essential for nurses’ resilience and retention. This process not only prevents breaches of the psychological contract but also promotes employee engagement and positive work experiences, which are crucial in reducing turnover intentions [71,72].

When exploring the impact of leader conscientiousness and ethical leadership on employee turnover intentions, Saleh et al. have focused on the mediating role of individual ethical climate [73]. The main results highlight the importance of leader conscientiousness (conscientious leaders are more likely to exhibit ethical leadership behaviors and act as role models, fostering trust and commitment among employees).

Ethical leadership appears negatively associated with turnover intentions [73], and recent research has further demonstrated that ethical leadership not only reduces turnover intention but also actual turnover in public institutions [74]. Moreover, recent findings in nursing contexts indicate that ethical leadership fosters compassion, enhances job performance, and contributes to professionals’ motivation and well-being [14]. That is, employees who work under the leadership of ethical leaders are less likely to leave their organizations. Conversely, ethical leadership positively impacts individual ethical climates (self-interest, friendship, and personal morality), which promotes a sense of fairness and professionalism.

Interestingly, ethical leadership reduces emotional exhaustion, a key factor in turnover intentions. A practical implication is to urge organizations to prioritize hiring and training conscientious leaders who can model ethical behavior.

Burnout, characterized by emotional exhaustion, depersonalization, and a decreased sense of personal accomplishment, is a significant predictor of turnover intentions among nurses [75].

Nurses are particularly vulnerable to burnout due to the demanding nature of their work, continuous patient contact, and the ongoing provision of care, often under stressful conditions [76]. The consequences extend beyond individual well-being, affecting job satisfaction and organizational commitment, ultimately compromising care quality and safety [77].

We recognize the ethical dimensions inherent in nursing practice [78,79], but little research has been done on the influence of ethical factors on nurse turnover intentions. It is within this context that moral resilience emerges as a potential strategy to mitigate the adverse consequences of burnout and subsequent turnover intentions [80].

Moral resilience can be defined as the ability to preserve or restore moral integrity in response to moral complexities, confusion, anguish, or setbacks in daily practice—and therefore, as the ability to navigate moral distress [81,82,83]. Consequently, it plays a crucial role in managing ethical dilemmas and reducing psychological distress.

It is also essential to emphasize the importance of moral courage in nursing practice, particularly among those in leadership roles. Moral courage refers to the inner strength to uphold ethical principles and act in accordance with one’s moral convictions, even when such actions may lead to personal or professional consequences [2].

For nurses, moral courage involves remaining faithful to the profession’s ethical standards and making decisions consistent with these values, even when doing so may result in criticism, conflict, or other forms of adversity [84,85]. Strengthening this quality is considered a key strategy to mitigate moral distress. Nurses who demonstrate higher levels of moral courage are better equipped to handle ethically complex situations, safeguard patients’ rights, and advocate for integrity in care delivery, all of which contribute directly to the overall quality of care [2,84,85,86].

Leadership exerts a significant influence on how nurses perceive and respond to ethical challenges in their workplace. Evidence showing a significant negative relationship between ethical leadership, moral distress, and turnover intention highlights the value of promoting this leadership style among nurse managers. Adopting ethically grounded leadership practices can therefore help to reduce both moral distress and the intention to leave the profession [50,87,88].

As Kenneth Blanchard and Vicent Peale assert, “There is no right way to do a wrong thing” (p. 19) [89] and the leader’s responsibility, the power of ethical management, stems precisely from being morally strong.

Nursing managers and hospital administrators should therefore implement measures that create supportive working environment for nurses and foster the development of moral courage among staff.

Nevertheless, we should reflect if people really desire an ethical leader. And why.

People desire ethical leaders because they inspire trust, foster a positive and engaged work environment, and contribute to long-term success through fairness, transparency, and accountability.

Ethical leadership fosters stronger relationships, ensures legal compliance, and ultimately contributes to a more resilient and successful organization. And it is hoped that people mirror their leader’s behaviour, embodying integrity, fairness, and accountability. Because, just as ethical education is done by example, leaders manage by example. The benefits are increased trust and engagement, a positive work environment, and inspire ethical behaviour.

Effective leaders do not politicize approaches for their own ends; they demonstrate deep respect for all. During the COVID-19 pandemic, it became clear that “a resilient society recognizes shared risks and institutionalizes shared security, especially for vulnerable groups. Justice has been at work in nations that have responded resiliently to COVID-19. Justice, not politics, forms the basis of what citizens owe to one another and what leaders owe to their citizens.” (Ruger, 2020) [67]. Leadership approaches, such as Quantum Leadership [90], emphasize adaptability, interconnection, and sustainability in complex systems, dimensions that resonate with healthcare organizations. As Porter-O’Grady and Malloch note, “Finding spirit in the chaos (…) gives leaders a firm center and inspires confidence and composure” (pp. 430–431) [90].

Finally, in a policy perspective, we take some ideas from Jennifer Ruger [66]. In the text “Health and Social Justice,” Ruger developed a theoretical model of justice for health, the health capability paradigm. Among the theoretical foundations of this model, we highlight the first—human flourishing and health capability, which are crucial for all people, above other merely instrumental social goods. Other foundations include the ethics of social determinants of health, which states that one should not attempt to improve health with broad policies that are not directly related to health, as the means and ends of health policies may become less clear, which may also limit our ability to assess the impact of public policies on health. Ultimately, the eight theoretical foundations of shared health governance facilitate the involvement of citizens, patients, health professionals, families, communities, insurers, and governments in developing consensus on substantive principles, distributive procedures, measures of effectiveness, standards, attitudinal changes, and deliberations to address health-related issues.

As a result of this shared governance, Ruger believes that equality of access is now viewed differently and should mean equal access to high-quality healthcare, not just minimal or adequate care.

## 3. Methodological Approach

This essay adopts a conceptual and reflective approach aimed at integrating ethical and organizational perspectives on workforce retention in healthcare. References were selected based on their relevance to the key themes of ethical climate, organizational justice, leadership, and professional well-being. Sources included peer-reviewed journal articles, books, and institutional reports published between 2010 and 2025, with priority given to works in English and Portuguese. Both theoretical and empirical studies were considered to support an integrated understanding of ethical and organizational dynamics. In addition, the analysis draws upon classical theoretical references, such as Lukács, Dejours, Rousseau, and Levinson, whose seminal contributions provide a philosophical and psychosocial foundation for understanding work, recognition, and the psychological contract. The literature reflects diverse healthcare contexts from Europe, North America, and Asia, emphasizing the global scope of retention and ethical challenges in health systems.

## 4. Discussion

This section synthesizes the theoretical insights developed throughout the essay, integrating the ethical and organizational perspectives that shape professional retention in healthcare. By connecting classical approaches to work and recognition with contemporary frameworks of ethical climate, organizational justice, and leadership, this discussion highlights the interdependence of moral well-being, professional commitment, and institutional sustainability.

Clinical practice environments are widely recognized as moral spaces because they are settings where ethical principles are applied, values are communicated, and the moral responsibilities of individuals are explored and enacted.

Moral spaces, in which institutional decisions shape professionals’ well-being, motivation, and intentions to stay or leave. Environments that provide structures, routines, and channels for discussing ethical obligations, influencing the moral behavior of professionals, and improving the quality of patient care.

Clinical settings present opportunities for ethical reflection and provide a platform for communicating and clarifying the ethical responsibilities that exist within healthcare institutions.

Supportive environments foster moral courage, enabling nurses to voice concerns and act ethically, even when facing personal or professional risks [70], as recent studies during the COVID-19 pandemic have demonstrated that organizational support plays a critical role in mitigating moral distress and reinforcing ethical practice.

A just culture, built on teamwork and open communication, can enhance moral courage and accountability. A truly ethical environment is inclusive, allowing individuals to express varying viewpoints without fear of reprisal.

Professional retention is not just an economic necessity or a staffing requirement. It goes much further, becoming an ethical imperative, a condition and requirement for the existence of competent professionals who provide safe, quality healthcare.

The very sustainability of the healthcare system is at stake, as high turnover fosters fragmentation, misaligns commitment, reduces team building, undermines respect for leadership, and undermines the sense of contextual development.

Organizational ethics intersect with clinical and social ethics when considering the mission and values of healthcare organizations. They should prioritize the well-being of professionals as much as their commitment to the quality of procedures and the brand’s advertising.

Retaining caregivers is not just a human resource management strategy but a collective ethical responsibility, with a direct impact on the quality of care and the sustainability of healthcare systems.

Within this framework, ethical leadership plays a central role in shaping and sustaining moral spaces at work. By demonstrating integrity and fairness, leaders help preserve the psychological contract between professionals and their organizations, thereby reinforcing the recognition and reciprocity that are essential foundations of trust and satisfaction. When practice environments are grounded in organizational ethics and supported by recognition, they not only reduce intentions to leave but also foster engagement, professional growth, and a stronger sense of belonging—factors that are critical to long-term retention.

Finally, professional retention should be understood as a collective ethical responsibility that goes beyond human resource management: it is an institutional and social obligation, as it directly impacts the quality of care delivered and society’s right to safe and sustainable healthcare services.

### 4.1. Practical and Managerial Implications

The conceptual synthesis developed in this essay carries several implications for healthcare organizations and managers. Recognizing professional retention as an ethical responsibility shifts the focus from purely administrative or economic solutions to the creation of moral work environments that sustain motivation, belonging, and commitment.

Managers play a central role in shaping these environments through ethical leadership, fairness, and recognition practices that reinforce psychological safety and professional meaning. Integrating ethics into management policies—such as recruitment, onboarding, continuous education, and evaluation—can foster resilience, reduce turnover, and enhance the quality of care.

From a broader perspective, this approach encourages healthcare systems to align human resource management with ethical governance principles, thereby promoting workforce sustainability and protecting the moral integrity of care delivery.

Ultimately, the contribution of this conceptual essay lies in reframing workforce retention as a strategic ethical imperative, bridging individual well-being, organizational justice, and the long-term sustainability of healthcare systems.

### 4.2. Limitations and Future Research

As a conceptual and reflective essay, this work does not rely on empirical data, which naturally limits its ability to quantify the relationships it discusses. However, this limitation is intrinsic to the chosen methodological approach, which seeks to generate theoretical integration and critical insight rather than statistical generalization.

The reflections presented here build on both classical and contemporary literature, offering a framework through which future empirical studies can examine the impact of ethical leadership, organizational justice, and recognition practices on professional retention.

Future research could empirically test the conceptual relationships proposed in this essay through mixed-method or longitudinal designs, focusing on how ethical and organizational variables interact to influence health professionals’ intention to remain in the profession. Comparative studies across different healthcare systems and cultural contexts would further deepen understanding of the ethical dimensions of workforce sustainability.

Despite its conceptual nature, this essay offers a structured foundation for empirical investigation by articulating ethical constructs with measurable organizational phenomena, thus bridging philosophy, ethics, and management science.

## 5. Conclusions

In conclusion, despite the growing attention to leadership in healthcare, significant gaps remain in the literature regarding the role of ethical leadership in nursing. Existing studies have primarily focused on job satisfaction, burnout, and organizational commitment; however, there is limited empirical evidence on how ethical dimensions—such as moral resilience, moral courage, or the ethical climate—interact with turnover intentions and nurse retention. This gap underscores the need for further research, particularly within nursing contexts, where ethical challenges are not abstract but are deeply embedded in daily practice, directly impacting both professional well-being and patient safety and outcomes.

From a practical and policy perspective, fostering ethical leadership in nursing should be recognized as not only a moral imperative but also a strategic organizational priority. Healthcare institutions should invest in ethics-oriented leadership training and in cultivating ethical climates, as these initiatives strengthen the psychological contract, reduce moral distress, and enhance professional engagement and retention. In doing so, organizations contribute simultaneously to the well-being of nurses and to the long-term sustainability, safety, and resilience of healthcare systems.

Looking forward, ethical leadership should be considered alongside other contemporary approaches to leadership. Quantum Leadership, for instance, with its emphasis on adaptability, interconnectedness, and value creation in complex and evolving systems, closely aligns with the realities of modern healthcare and nursing practice. Although not the primary focus here, these models converge with ethical leadership in underscoring the importance of ethics, resilience, and professional well-being. Bringing these perspectives together can foster healthier, fairer, and more supportive workplaces, where nurses are encouraged to thrive and to remain in the profession.

Ultimately, retaining healthcare professionals cannot be viewed solely as a managerial or financial issue. It is, above all, the collective ethical responsibility of organizations and of society. The stability of those who provide care is inseparable from delivering safe, high-quality, and sustainable healthcare for everyone.

## Data Availability

No new data were created or analyzed in this study. Data sharing is not applicable to this article.

## Abbreviations

The following abbreviations are used in this manuscript:

OECD	Organisation for Economic Co-operation and Development
